# Retinocytoma: A Case Series

**DOI:** 10.7759/cureus.42958

**Published:** 2023-08-04

**Authors:** Luluah Bubshait, Khalid Alburayk, Halla Alabdulhadi, Khalid Emara

**Affiliations:** 1 Department of Ophthalmology, College of Medicine, Imam Abdulrahman Bin Faisal University, Dammam, SAU; 2 Department of Ophthalmology, Ministry of Health, Riyadh, SAU; 3 Cornea and Anterior Segment Division, King Khaled Eye Specialist Hospital, Riyadh, SAU; 4 Department of Ophthalmology, Pediatric Ophthalmology Division, Dhahran Eye Specialist Hospital, Dhahran, SAU

**Keywords:** pediatric, retina, tumer, retinoblastoma, retinocytoma

## Abstract

Retinoblastoma is the most common ocular malignancy in children, considered fatal without treatment. Retinocytoma is a rare benign clinical entity of retinoblastoma that shows signs of tumor regression. The clinical presentation of retinocytoma usually includes a gray translucent mass, intralesional calcification, and retinal pigment epithelial alteration, along with the presence of chorioretinal atrophy. We report two cases of retinocytoma in patients presenting with strabismus in the Eastern Province of Saudi Arabia.

## Introduction

Retinocytoma is a rare benign retinal tumor that is generally considered a non-progressive variant of retinoblastoma [1]. It usually presents as one or more retinal masses that are gray and translucent in color, along with calcific nodules and retinal pigment epithelial changes, which resemble retinoblastoma regression after treatment [1]. Due to this clinical resemblance, other names such as "spontaneously regressed retinoblastoma", "retinoblastoma group 0", and "retinoma" have been used interchangeably [2]. In such cases, the diagnosis cannot be confirmed histopathologically; however, clinical diagnosis by experts in the field can be relied upon. Furthermore, mitosis and necrosis are typically absent in retinocytoma [3]. Careful diagnosis of retinocytoma must be made by identifying the pathognomonic clinical findings to differentiate it from retinoblastoma, as it usually requires close observation rather than aggressive management [4].

Only a few case reports and series have been reported in the literature [2,5,6]. To the best of our knowledge, one case series of retinocytoma was reported in the Middle East. Here, we present two cases of retinocytoma in the Eastern Province of Saudi Arabia.

## Case presentation

Case presentation 1

**Figure 1:**
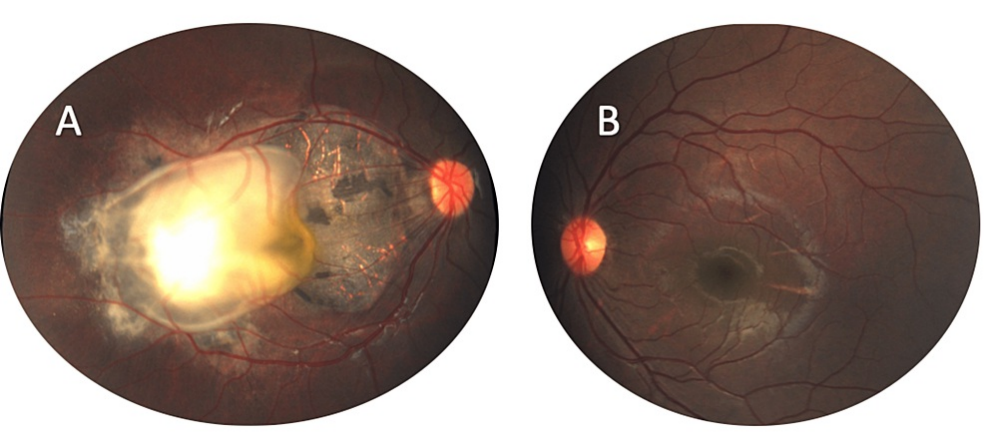
Color fundus photos of the right eye with retinocytoma (A) and the left eye with normal fundus (B).

**Figure 2:**
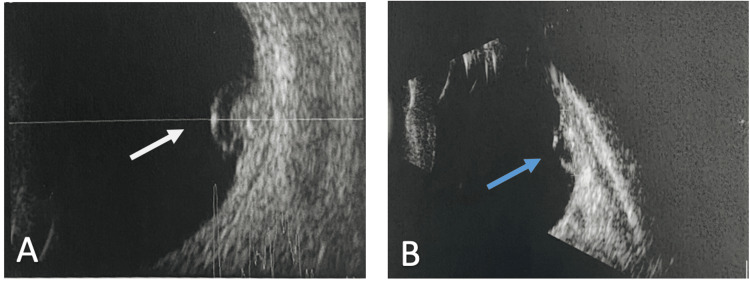
Ocular ultrasound of the right eye showing the retinocytoma (white arrow) at the time of presentation (A) and the mass two years (blue arrow) later (B).

A five-year-old healthy boy with a history of right esotropia for the past three years was referred to our facility after a retinal mass was identified during a routine ophthalmic examination and cycloplegic refraction at another location. He has no significant medical, surgical, or family history. On examination, he presented with 20/200 vision in the right eye and 20/20 vision in the left eye. The cover test revealed right esotropia, but he had full ocular motility. Pupils were round, regular, and reactive, with no relative afferent pupillary defect. The anterior segment examination of both eyes was completely normal. During the fundus examination of the right eye, a 5 x 5 mm greyish-whitish calcific macular tumor surrounded by subretinal fluid and retinal pigment epithelial changes was observed (Figure 1-A). However, the fundus examination of the left eye was completely normal (Figure 1-B). Ultrasonic examination (A-mode) of the right eye showed medium to low internal reflectivity, while B-mode revealed clear vitreous, an elevated lesion at the macular area, and areas of hyper- and hypo-echogenicity representing calcifications and subretinal fluids, respectively. The optic nerve head and choroid appeared normal (Figure 2-A). The intraocular pressure was measured to be 12 mm Hg and 13 mm Hg in the right and left eye, respectively. A careful diagnosis of retinocytoma was provided by an experienced ocular oncologist at our facility based on the typical ophthalmoscopic and ultrasonic findings. Therefore, the patient was placed under observation. Upon follow-ups, there were no signs of progression of the tumor in terms of its size. The lesion showed stability till the last follow-up three years after presentation (Figure 2-B). 

 

Case presentation 2 

**Figure 3:**
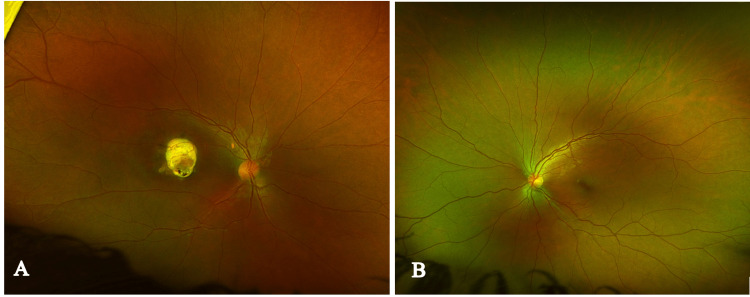
Color fundus photos of the right eye (A) with retinocytoma and the left eye (B) with normal fundus.

A 24-year-old healthy female presented to our facility with a history of poor vision in her right eye since childhood, which was associated with exotropia for the past two years. She had no significant medical, surgical, or family history. During the examination, her visual acuity was measured as 20/400 in the right eye and 20/20 in the left eye. The cover test revealed right exotropia with full ocular motility. Pupils were round, regular, and reactive, but a relative afferent pupillary defect was observed in her right eye. The anterior segment examination of both eyes was completely normal. Upon fundus examination of the right eye, a whitish elevated dome-shaped lesion measuring 3.5 x 3.5 mm with retinal pigment epithelium atrophy and a flat retina were observed (Figure 3-A). However, the fundus examination of the left eye was completely normal (Figure 3-B).

**Figure 4:**
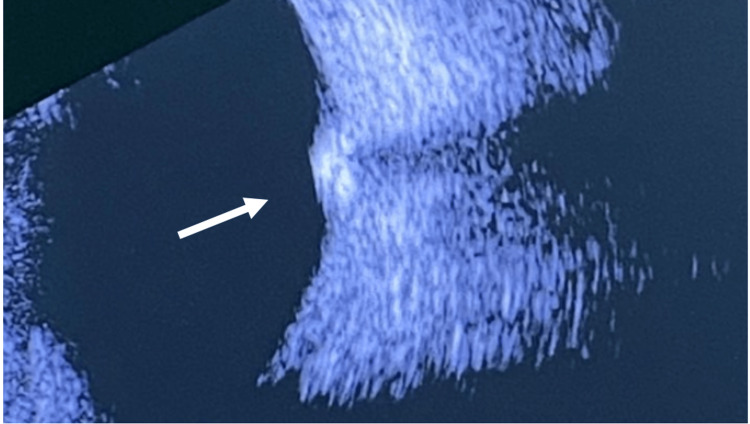
Ocular ultrasound of the right eye showing retinocytoma (white arrow).

Ultrasonic examination (B-mode) of the right eye showed clear vitreous and revealed an elevated lesion at the macular area with areas of hyperechogenicity representing calcifications. The optic nerve head and choroid appeared normal (Figure 4). The intraocular pressure was measured to be 19 mm Hg in both eyes. A careful diagnosis of retinocytoma was made based on the typical ophthalmoscopic and ultrasonic findings. Upon serial observations and follow-ups spanning over 10 years, the retinal mass displayed significant regression in size, with chorioretinal atrophy surrounding it.

## Discussion

The term "retinoma" was first introduced by Gallie et al. in 1982 to describe a non-progressive retinal lesion in patients carrying the retinoblastoma gene [4]. The incidence of spontaneous regression of retinoblastoma cases varies in the literature, with reported rates ranging from 0.04% in Saudi Arabia [7] to 9.0% in Chinese reports and 5.3% in Taiwan reports [6,8]. In our facility, the incidence of retinoblastoma spontaneous regression was found to be 1%.

Most patients with retinocytoma are asymptomatic and are diagnosed either through routine eye examinations or when a family member is diagnosed with retinoblastoma, prompting eye exams for the rest of the family [9]. Clinical presentation of retinocytoma typically includes a gray translucent mass, intralesional calcification, retinal pigment epithelial alteration, and the presence of chorioretinal atrophy [4].

Despite the unique ophthalmoscopic features described above, certain entities such as retinoblastoma, astrocytic hamartoma, and myelinated nerve fibers may mimic retinocytoma [9]. Traditionally, retinocytoma is considered a benign tumor that does not require treatment. However, it is important to note that retinocytoma does not guarantee a stationary course. While very few cases have reported the transition of retinocytoma to a malignant state, there have been instances of lethal complications due to chiasmatic infiltration resulting from the transformation of retinocytoma to retinoblastoma [10]. According to Singh et al., there is a 4% rate of retinocytoma transitioning to malignancy. Therefore, lifelong follow-up of patients with retinocytoma is essential to prevent progression to retinoblastoma [2].

The development of retinoblastoma, as described in the two-hit model, requires both alleles. The first hit usually occurs in the germline, either inherited from one parent or acquired early in embryonic development. The second hit is typically a somatic mutation that occurs after zygote formation [11]. However, despite the common genetic implications shared by retinocytoma and retinoblastoma, it remains unclear why some individuals develop retinocytoma rather than retinoblastoma. Hypothetically, retinocytoma may result when the second hit occurs later during cell development and maturation, at a stage when the originator cell has minimal mitotic potential [12]. Another hypothesis suggested by Dryja et al. proposes that retinocytoma may be a result of low penetrance retinoblastoma [13].

## Conclusions

We described the clinical features of two patients who presented to our facility with retinocytoma. A careful diagnosis was made based on typical ophthalmoscopic and ultrasonic findings. The incidence of retinocytoma cases in our hospital was found to be 1%. Although the lesions showed no signs of progression, regular long-term follow-ups are crucial due to their rare possibility of transformation into retinoblastoma.

## References

[1] Aaby AA, Price RL, Zakov ZN (1983). Spontaneously regressing retinoblastomas, retinoma, or retinoblastoma group 0. Am J Ophthalmol.

[2] Singh AD, Santos CM, Shields CL, Shields JA, Eagle RC Jr (2000). Observations on 17 patients with retinocytoma. Arch Ophthalmol.

[3] Margo C, Hidayat A, Kopelman J, Zimmerman LE (1983). Retinocytoma: a benign variant of retinoblastoma. Arch Ophthalmol.

[4] Gallie BL, Ellsworth RM, Abramson DH, Phillips RA (1982). Retinoma: spontaneous regression of retinoblastoma or benign manifestation of the mutation?. Br J Cancer.

[5] Lam A, Shields CL, Manquez ME, Shields JA (2005). Progressive resorption of a presumed spontaneously regressed retinoblastoma over 20 years. Retina.

[6] Kao LY, Yang ML (2005). Spontaneous regression of retinoblastoma in a Taiwan series. J Pediatr Ophthalmol Strabismus.

[7] Al-Mesfer S, Souru C, Khandekar R (2020). Clinical profile of spontaneously regressed retinoblastoma tumours. Arch Clin Med Case Rep.

[8] Xu X, Li B, Wang Y (2014). Retinoblastoma spontaneous regression: clinical and histopathologic analysis (Article in Chinese). Chin J Ophthalmol.

[9] Bowen R, Stathopoulos C, Munier F, Singh AD (2019). Retinocytoma or retinoma. Clinical Ophthalmic Oncology.

[10] Mataftsi A, Zografos L, Balmer A (2012). Chiasmatic infiltration secondary to late malignant transformation of retinoma. Ophthalmic Genet.

[11] Harbour JW, Dean DC (2000). Rb function in cell-cycle regulation and apoptosis. Nat Cell Biol.

[12] Gallie BL, Dunn JM, Chan HS, Hamel PA, Phillips RA (1991). The genetics of retinoblastoma: relevance to the patient. Pediatr Clin North Am.

[13] Dryja TP, Rapaport J, McGee TL, Nork TM, Schwartz TL (1993). Molecular etiology of low-penetrance retinoblastoma in two pedigrees. Am J Hum Genet.

